# Characteristics of ADHD Symptom Response/Remission in a Clinical Trial of Methylphenidate Extended Release

**DOI:** 10.3390/jcm8040461

**Published:** 2019-04-05

**Authors:** Margaret Weiss, Ann Childress, Earl Nordbrock, Akwete L. Adjei, Robert J. Kupper, Greg Mattingly

**Affiliations:** 1 Child and Adolescent Psychiatry, Cambridge Health Alliance, Cambridge, MA 02138, USA; 2 Center for Psychiatry and Behavioral Medicine, Inc., Las Vegas, NV 89128, USA; drann87@aol.com; 3 Rhodes Pharmaceuticals L.P., Coventry, RI 02816, USA; earlnordbrock@aol.com (E.N.); akwete.adjei@rhodespharma.com (A.L.A.); Robert.kupper@rhodestec.com (R.J.K.); 4 Washington University School of Medicine, Washington University, St. Louis, MO 63110, USA; greg@mattingly.com; 5 Midwest Research Group, St. Charles, MO 63304, USA

**Keywords:** attention deficit/hyperactivity disorder, central nervous system stimulants, methylphenidate, remission, response

## Abstract

Clinical trials in attention-deficit/hyperactivity disorder (ADHD) have typically measured outcome using clinician ratings on the Attention-Deficit/Hyperactivity Disorder Rating Scale, Fourth Edition (ADHD-RS-IV) and the Clinical Global Impression-Improvement (CGI-I) scale. Remission has been defined as an endpoint score of less than or equal to 18 on the ADHD-RS-IV (or a mean score of 1). Responders have been defined as patients who achieve a CGI-I score of much or very much improved (1 or 2). There is a lack of agreement in the literature on what percent change in symptoms on the ADHD-RS-IV should be used to define improvement or remission. This study uses data from a clinical trial of a methylphenidate extended release (MPH-MLR; Aptensio XR^®^) phase III clinical trial to attempt to determine the percent change of symptoms that best corresponds with improvement and remission. Symptom remission at endpoint (ADHD-RS-IV total score ≤18) was most closely aligned with a ≥46% reduction in ADHD-RS-IV total score. Clinical improvement was most closely aligned with a ≥40% reduction in ADHD-RS-IV total score. The three different measures of outcome were strongly aligned during double blind and open label treatment, and were independent of subtype status. Our data suggest that at least 40% improvement in symptoms is needed to achieve a robust response at endpoint.

## 1. Introduction

Clinical trials of medication and psychological treatments for attention-deficit/hyperactivity disorder (ADHD) have looked at outcome in terms of symptom change from baseline, overall clinical improvement, and final symptom endpoint. Each of these provides a slightly different perspective on treatment response. For example, percent decrease in symptom scores reflects change from baseline. Clinical global impression of improvement also reflects change from baseline, but goes beyond core symptoms. Remission reflects patient achievement of a final outcome that would be considered “normalization”. Endpoint scores are distinct from change scores in that if someone starts treatment very ill (i.e., a symptom score of 54) and has a 50% drop in symptoms, they may still be moderately symptomatic. Past trials have used arbitrary cutoffs for percent change in symptoms ranging from 25% to 40%. 

The importance of differentiating response in terms of improvement vs. remission first became apparent in a post hoc reanalysis of the Multimodal Treatment Study of Children with ADHD study [1]. Differences between the treatment arms that were not obvious looking solely at improvement were much more apparent when considering remission or normalization [2]. Biederman et al. [3] further defined three standards of outcome that can be applied to age-dependent decline in ADHD: syndromatic (less than full syndrome), symptomatic (less than subthreshold diagnosis), and functional (full recovery) remission. This study was unique; the authors compensated for the impact of subtype on final score by looking at three symptom clusters independently: attention, hyperactivity, and impulsivity. They defined “syndromatic remission” as failing to meet the full diagnostic criteria for ADHD, which meant the patient still had symptoms but no longer met diagnostic criteria. “Symptomatic remission” required that the patient have fewer than the number of symptoms required for a subthreshold diagnosis. The most robust definition of remission was “functional remission”, which required that the patient have fewer than 36% of the symptoms of ADHD and no impairment (score on the Global Assessment of Functioning Scale higher than 60). Few clinical trials have followed this methodology in distinguishing not just symptom improvement from symptom remission, but also in looking at remission as requiring both normalization of symptoms and functioning. Even more important in this study was the use of a methodology that looked at attention, hyperactivity and impulsivity independently [3]. In a patient with combined type ADHD, all 18 items are equally relevant; however, in a patient with only attention problems, the total score underestimates severity, because only the 9 items of attention are relevant. 

Throughout the various versions of the Diagnostic and Statistical Manual of Mental Disorders (DSM), rating scales for ADHD have scored the 18 DSM symptoms of ADHD on a 4-point Likert scale. The anchor points for measurement of symptoms have been either based on frequency (not at all = 0, just a little = 1, pretty much = 2, or very much = 3, such as the Swanson, Nolan, and Pelham Questionnaire [SNAP] [2]) or severity (never = 0, occasionally = 1, often = 2, or very often = 3, such as the Vanderbilt [4]). We are not aware of any studies comparing the relative validity or relationship between using frequency or severity ratings. A consensus standard in the field, however, has been to consider any rating of 2 or 3 as symptomatic, whether this is defined by severity or frequency. A symptom rated 2 or 3 is considered to be present, and whether or not a patient meets the criteria for syndromatic remission is then determined by whether they meet the 6/9 criteria for attention or hyperactive/disruptive symptoms (or 5/9 if older than 17 years). 

The Attention-Deficit/Hyperactivity Disorder Rating Scale, Fourth Edition (ADHD-RS-IV) rates each of the 18 DSM, Fourth Edition (DSM-IV) ADHD symptoms as never or rarely = 0, sometimes = 1, often = 2, and very often = 3 [5]. The DSM-IV defines mild ADHD as few symptoms in excess of those required for diagnosis and mild impairment; and severe ADHD as many symptoms in excess of those required to make the diagnosis, symptoms that are very severe, or marked impairment. It should be noted, however, that normative studies have defined T scores that show considerable variation between ages and genders, although T scores are rarely used in outcome analysis. An ADHD-RS-IV total score ≤18 (i.e., mild or less on average) has been used as a cut point for ADHD symptomatic remission [6]. 

A wide range of definitions and assessment tools have been used to try to define treatment response and disease remission; medication trials in children have used a variety of endpoints to describe these terms [7,8,9,10,11]. This is the first study we are aware of that attempts to look empirically at what percent change in symptoms is best anchored to global improvement and remission. In this secondary analysis, data from a large phase III methylphenidate multi-layer release (MPH-MLR) trial (ClinicalTrials.gov identifier NCT01239030) were utilized to investigate various measures of clinical response and symptomatic remission associated with MPH-MLR treatment in children and adolescents with ADHD.

MLR-MPH is a once-daily beaded formulation composed of an immediate-release layer and a delayed-release layer. Administration results in a rapid rise in plasma levels after oral administration with an onset of action within 1 h and a duration of action of 12 h. MLR-MPH has a bimodal release such that the outer layer is equal to that of immediate-release MPH with initial rapid dissolution and absorption of 40% of the dose, followed by an evenly maintained release of MPH through the day from the second layer.

The relationship between percent change in the ADHD-RS-IV total score versus a predefined symptom threshold cutoff of ADHD-RS-IV total score ≤18 (symptomatic remission) versus a Clinical Global Impression (CGI)-Improvement (CGI-I) score of much or very much improved was explored to further understand the relationship and agreement between these outcomes. Furthermore, whether these relationships are similar for the combined versus inattentive ADHD subtypes will be explored. There were not enough patients with hyperactive/impulsive subtype to power inclusion of this group.

## 2. Materials and Methods

### 2.1. Trial Design

The design and primary results of this phase III, randomized, double-blind, placebo-controlled, parallel, fixed-dose study of MPH-MLR safety and efficacy have been previously published [12]. Briefly, the study included 4 phases: screening (≤28 days), double-blind fixed-dose phase (1 week), open-label dose-optimization phase (11 weeks), and a follow-up call. Children and adolescents (male and female) aged 6–18 years at the time of consent with an ADHD diagnosis of all subtypes (except “not otherwise specified”) as defined in the DSM-IV, Text Revision were included. During the double-blind fixed-dose phase, patients were randomized (1:1:1:1:1) to receive MPH-MLR 10, 15, 20, or 40 mg, or placebo. During the open-label dose-optimization phase, treatment was initiated with a once-daily MPH-MLR 10 mg dose, unless the investigator determined, from earlier experience, that a higher dose would be needed, and titrated (up or down) at weekly intervals until dosing was optimized. The dose was increased until there was no further improvement in the ADHD-RS or CGI or further increases were limited by side effects. There was an option to go beyond 40 mg to 60 mg if required. At completion of the open-label dose-optimization phase, patients were offered the opportunity to continue to receive MPH-MLR for up to 21 months (open-label compassionate use extension) for those patients requiring ongoing treatment. The length of the open-label compassionate use extension was modified during the study, and thus not all patients had the full 21 months available to them. 

This study was conducted in compliance with the Good Clinical Practice guidelines of the International Conference on Harmonization of Technical Requirements for Registration of Pharmaceuticals for Human Use, the US Code of Federal Regulations that relate to clinical trial conduct, and the principles of the Declaration of Helsinki. An institutional review board for each of the 16 study sites reviewed and approved the protocol, amendments, and informed consent.

### 2.2. Measures

#### 2.2.1. Attention-Deficit/Hyperactivity Disorder (ADHD) Symptom Change

ADHD symptoms were measured using the ADHD-RS-IV [4]. The ADHD-RS-IV has acceptable psychometric properties including inter-rater reliability, test-retest reliability, internal consistency, factor structure, convergent and divergent validity, discriminant validity, and responsiveness. The scale was administered by trained clinicians at baseline (start of the double-blind fixed-dose phase [visit 2]), at the end of the double-blind fixed-dose phase on day 7 (visit 3), and on days 14 (visit 4), 21 (visit 5), 28 (visit 6), 56 (visit 7), and 84 (visit 8), and throughout the open-label compassionate use extension (visits 15–18; visits 15–18 were nominally every 2 months, and few patients were enrolled after visit 18, although some continued for up to 21 months). Each item on the ADHD-RS-IV is reflective of 1 of the 18 ADHD symptoms included in the DSM-IV. 

#### 2.2.2. Clinical Global Impression (CGI)

The CGI is a measure that is used as a standard in clinical trials to reflect the clinician’s initial global impression of patient severity (CGI-S) at baseline and improvement with treatment (CGI-I). The CGI-S [13] is a 7-point scale that requires the clinician to rate the severity of the patient’s illness at the time of assessment, relative to the clinician’s past experience with patients who have the same diagnosis. Possible ratings range from normal to among the most severely ill patients. The CGI-I is a 7-point scale that requires the clinician to assess how much of the patient’s illness has improved or worsened relative to a baseline state at the beginning of the intervention and rated as: very much improved, much improved, minimally improved, no change, minimally worse, much worse or very much worse. By convention, the CGI-I is analyzed as a categorical variable reporting the percent of patients at endpoint who are rated as much or very much improved. In this trial the CGI-I score was rated by the clinician based on all data at day 7 (end of the double-blind fixed-dose phase); and on days 14, 21, 28, 56, and 84; and throughout the open-label compassionate use extension. 

#### 2.2.3. Remission

Remission has been variously defined as a mean score of ≤1 of all 18 items [2] on the ADHD-RS-IV or as a final endpoint score of ≤18. For the purposes of this study, remission was defined as a final score of ≤18 [14], which is the definition of remission accepted in the literature. 

### 2.3. Analyses

This post hoc analysis determined the percentage reduction in ADHD-RS-IV total score that best aligned with CGI-I score ≤2 (very much or much improved). For each visit after baseline, the percentage reduction in ADHD-RS-IV total score was calculated as:

100 × (baseline visit score − this visit score)/baseline visit score

The percentage reduction that best aligned with CGI-I score ≤2 was determined as follows. For each visit, for each percentage reduction (ranging from 30–50%), the percentage of patients with at least the given reduction was compared with the percentage of patients with CGI-I score ≤2, and the percentage of patients with agreement between the 2 measures was calculated. The range was derived from the consensus that 30% has been described as the lower limit defining improvement, and 50% as the upper limit defining remission. For example, perfect agreement for a reduction of ≥30% and CGI-I score ≤2 would occur if all patients with a reduction of ≥30% had CGI-I score ≤2 and all patients with a reduction of <30% had CGI-I score >2. The 95% confidence interval (CI) for the percentage of patients with agreement was calculated, and McNemar’s exact test was used to compare the percentage of patients with at least the given reduction to the percentage of patients with CGI-I score ≤2. The percentage reduction that best aligned was the one with the greatest percentage of patients with agreement. Fisher’s exact test was used to compare the combined and inattentive subtypes using the percentage of patients with agreement of combined and inattentive ADHD subtypes.

This post hoc analysis also determined the percentage reduction in ADHD-RS-IV total score that best aligned with ADHD-RS-IV total score ≤18 with a similar method.

All calculations utilized SAS 9.4 (SAS Institute Inc., Cary, NC, USA).

## 3. Results

### 3.1. Response to Treatment

At least 1 dose of the study drug was taken by 230 enrolled patients (safety population), 221 patients were included in the evaluable population, and 200 patients completed the open-label dose-optimization phase. Two-thirds of the sample were stimulant naïve, and one-third of the sample (75/221) had a medication washout. The option to enter the open-label compassionate use extension was exercised by 173 patients, with 128, 112, 100, and 66 still in the study after visits 15, 16, 17, and 18, respectively. The open-label compassionate use extension encompassed up to 21 months of treatment. Combined was the most common ADHD subtype (Table 1).

**Figure 1:**
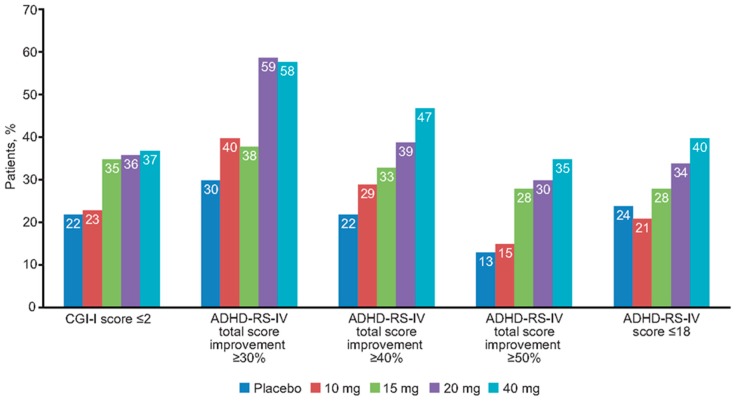
Percentage of patients achieving clinical response or symptomatic remission after 1 week of double-blind fixed-dose methylphenidate extended-release treatment. ADHD-RS-IV: Attention-Deficit/Hyperactivity Disorder Rating Scale, Fourth Edition; CGI-I: Clinical Global Impressions-Improvement.

After 7 days of double-blind fixed-dose treatment with no initial dose titration, 45% of the 221 patients achieved ≥30% symptomatic improvement, 34% achieved ≥40% symptomatic improvement, 24% achieved ≥50% symptomatic improvement, 30% had clinical response as defined by CGI-I score ≤2, and 29% were in remission with an ADHD-RS-IV total score ≤18. In general, the degree of clinical response and symptomatic remission achieved at the end of the 1-week double-blind fixed-dose phase was highly dose related, with symptomatic remission ranging from 21% at a dose of 10 mg daily to 40% at a dose of 40 mg daily (Figure 1). 

### 3.2. Relationship between Percent Change in Symptoms and Clinical Global Improvement at End of Double-Blind Treatment

At double-blind fixed-dose treatment end, a CGI-I score ≤2 was best aligned with a 40% reduction in ADHD-RS-IV total score, with CGI-I score ≤2 in 30% of patients and a ≥40% reduction in ADHD-RS-IV total score in 34% of patients, and the percentage of patients was not statistically different for these 2 measures (McNemar’s exact test, *p* = 0.26). Agreement between these measures was evident in 87% of patients (192/221; 95% confidence interval (CI), 82–91%). Percentage of patients with agreement was similar for combined (117/134 [87%]) and inattentive (63/72 [88%]) subtypes (Fisher’s exact test, *p* = 1.00). 

**Figure 2:**
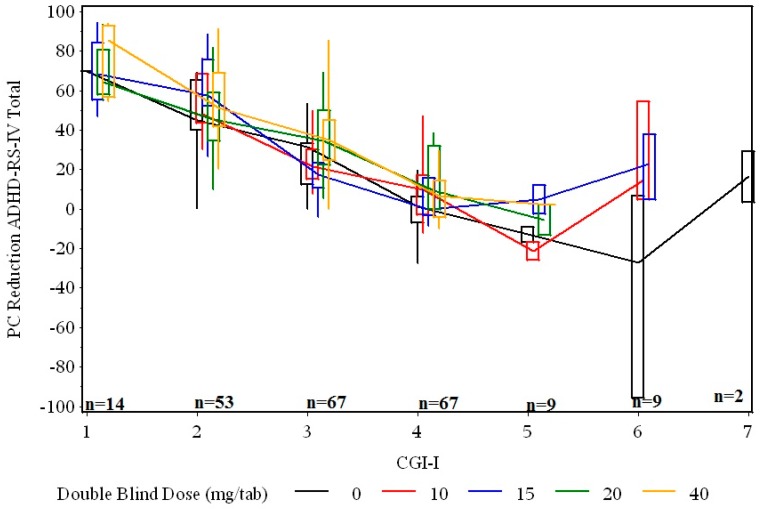
ADHD-RS-IV Total score percent reduction vs CGI-I after 1 week of double-blind fixed dose. The lines connect the medians. The box is the 25^th^ to 75^th^ percentile. The whiskers extend to the extremes.

Figure 2 plots the percent reduction in symptoms vs. CGI-I, for each dose. There is a wide range for values for both CGI-I and ADHD-RS-IV Total score improvement. Moreover, the relationship appears to be similar for all dose groups.

### 3.3. Relationship between Percent Change in Symptoms and Remission Outcome at End of Double-Blind Treatment

At the end of double-blind fixed dose treatment, remission (ADHD-RS-IV total score ≤ 18) most closely aligned with a 46% reduction in ADHD-RS-IV total score, with remission in 29% of patients and a 46% reduction in ADHD-RS-IV total score in 27% of patients, and the percentage of patients was not statistically different for these 2 measures (McNemar’s exact test, *p* = 0.56). There was agreement between these measures in 88% of patients (195/221; 95% CI, 83–92%). The percentage of patients with agreement was similar for combined (120/134 [90%]) and inattentive (61/72 [85%]) subtypes (Fisher’s exact test, *p* = 0.37).

### 3.4. Relationships at End of Open-Label Dose Optimization Phase

**Figure 3:**
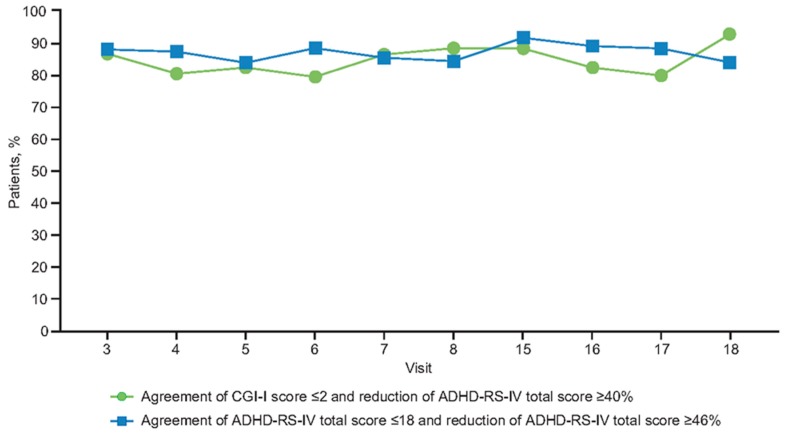
Percentage of patients with agreement between clinical response (CGI-I score ≤ 2) and a ≥40% reduction in ADHD-RS-IV total score, and the percentage of patients with agreement between an ADHD-RS-IV total score ≤18 and a ≥46% reduction in ADHD-RS-IV total score. Visits 15–18 were nominally every 2 months after completion of the open-label dose-optimization phase. ADHD-RS-IV: Attention-Deficit/Hyperactivity Disorder Rating Scale, Fourth Edition; CGI-I: Clinical Global Impressions-Improvement.

**Figure 4:**
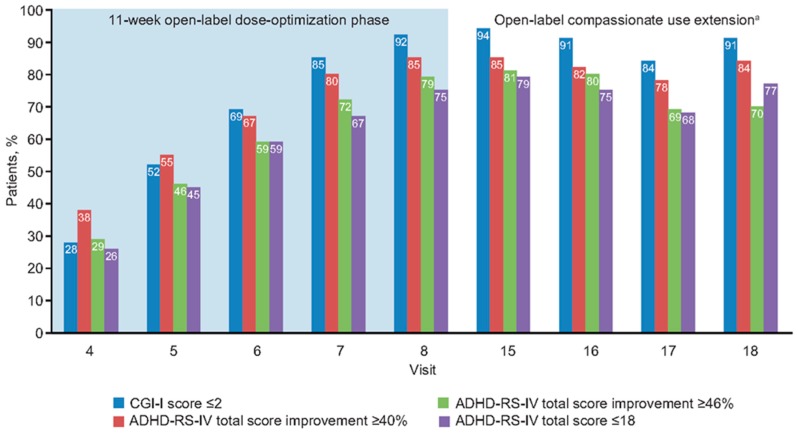
Improvement in different attention-deficit/hyperactivity disorder measures with open-label methylphenidate extended-release treatment. ^a^ Visits 15–18 were nominally every 2 months after completion of the open-label dose-optimization phase. ADHD-RS-IV: Attention-Deficit/Hyperactivity Disorder Rating Scale, Fourth Edition; CGI-I: Clinical Global Impressions-–Improvement.

At the end of the 11-week open-label dose-optimization phase, most patients had responded to treatment; 94% (188/200), 85% (170/200), and 73% (146/200) of patients receiving MPH-MLR had a ≥30%, ≥40%, and ≥50% reduction in ADHD-RS-IV total score, respectively. Clinical response, defined as a CGI-I score ≤2, was achieved in 92% (183/200) of patients. Symptomatic remission (ADHD-RS-IV total score ≤18) was achieved in 75% of patients. Throughout the open-label dose-optimization phase, CGI-I score ≤2 remained closely aligned, with a 40% reduction in ADHD-RS-IV total score (Figure 3 and Figure 4). At the end of the open-label dose-optimization phase, agreement between CGI-I score ≤2 and a ≤40% reduction in ADHD-RS-IV total score was evident in 89% of patients (177/200; 95% CI, 83–93%). Throughout the open-label dose-optimization phase, symptom remission (ADHD-RS-IV total score ≤18) remained closely aligned with a 46% reduction in ADHD-RS-IV total score (Figure 3 and Figure 4). At the end of the open-label dose-optimization phase, agreement between remission and a 46% reduction in ADHD-RS-IV total score was evident in 85% of patients (95% CI, 79–89%). Percentage of patients with agreement was similar for combined (107/122 [88%]) and inattentive (60/66 [91%]) subtypes (Fisher’s exact test, *p* = 0.63).

### 3.5. Relationships During Open-Label Compassionate Use Extension

Throughout the open-label compassionate use extension, overall response and remission rates were maintained by the majority of patients. There was sustained alignment between symptomatic response (≥30% symptom improvement in ADHD-RS-IV total scores) and the clinician’s global assessment of being much or very much improved, with CGI-I scores ≤2 (Figure 3 and Figure 4). Symptom improvement of ≥46% remained in agreement with symptomatic remission.

## 4. Discussions

Throughout various phases of the study, the percentage of patients achieving 40% symptom improvement was in best agreement with achieving CGI-I response. A 46% symptom improvement was in best agreement with symptom remission. While ~90% of patients in this trial were able to achieve some symptom improvement (defined as ≥30% improvement in ADHD-RS-IV total score), it should be noted that only ~75% were able to achieve symptom remission. This pattern of response, in which most patients achieve improvement and a majority achieve remission, is consistent with other stimulant trials [15,16,17]. 

These data help to clarify our understanding of the relationship between symptom change, clinical improvement and symptom remission. Although each of these outcomes describes a slightly different way of looking at response, this study demonstrates that these outcomes are closely aligned. Our results emphasize the importance of targeting remission rather than simply response as the standard of care. Most important, this study provides empirical criteria to define the percent of change that should be considered as the convention for response in clinical trials. Previous definitions of symptom response that used 30% change as an acceptable measure of outcome probably were generous in identifying whether patients showed some degree of palliation with intervention, but may have contributed to clinicians failing to treat to optimization. Further research needs to look at how these definitions of symptom response correlate with actual functional improvement and remission. 

Importantly, analyses of data from this pivotal trial demonstrated that the agreement of CGI-I score ≤2 and a 40% reduction in ADHD-RS-IV total score held for patients with combined and predominantly inattentive subtypes. The agreement between and ADHD-RS-IV total score ≤18 and a ≥46% reduction in ADHD-RS-IV total score also held for both of these subtypes. This likely reflects the fact that the majority of patients in the trial had challenges in both domains. Clinicians should be cautious in looking only at total scores for 18 items, when looking at outcome in individuals where problems are largely limited to either attention or hyperactive/impulsive difficulty. For example, if a patient has a mean score of 2 for 9 symptoms of just hyperactive or just inattentive symptoms, they would be clinically moderately ill, but would still meet the criteria for “remission” defined as an endpoint score of 18.

A robust standard for fine tuning a high standard of treatment response, as per the methodology used by Swanson in comparing treatments in the Multimodal Treatment of ADHD (MTA) study [2], would determine the number of patients with full symptomatic and functional response. While this might reveal cross-treatment differences that are not otherwise apparent, even more important is that setting the bar to this standard would encourage clinicians to look at optimizing dose, and augmenting medication with environmental and psychosocial interventions to treat residual difficulties which would further improve the lives of patients with ADHD. 

This study has several limitations. This is an exploratory post hoc analysis using data from a pivotal trial that included a representative ADHD patient population and used a phase III, double-blind, parallel, fixed-dose, randomized design, followed by open label. Given the conventional methodology of the trial, this is a reasonable sample with which to examine the relationship between outcome and measures. Nonetheless, the results are representative of the particulars of this design and patient population and need to be replicated in other types of trial design, populations, and types of treatment intervention. Two-thirds of the sample were stimulant naïve, and it is unclear how this may have impacted patient perception of symptoms. Although we had a full range of responses, partly because the double-blind period was short and dose was randomized rather than optimized, the high agreement between outcomes we obtained in open-label follow-up may reflect a limitation of the high rate of response in open label follow ups, given that non-responders would not typically stay in the study. It should also be noted that all of these measures are completed by clinicians and have a certain degree of overlap. For example, the clinician is aware of the ADHD-RS rating when completing the CGI, so our results do not reflect truly independent measures or interrater reliability. No special training was done to standardize clinician ratings, but again this is not atypical of clinical trials conduct. Nonetheless, this is how these outcomes are completed in clinical trials in ADHD and reflect real world practice. Further research on standardizing appropriate clinical trial outcomes in ADHD, avoiding redundancy between scale outcomes that overlap, and empirical definition of response vs. remission are needed. This research would require receiver operating characteristics to determine the most sensitive cut points, and logistic regression analysis to compare across measures and outcomes. 

Other limitations of this study reflect limitations of current methodology for measuring outcome in ADHD clinical trials in general. First, although by definition remission should be considered as per the work of Biederman et al. [3] as both symptom and functional remission, the definition used here refers only to symptoms. 

## 5. Conclusions

This is the first study we are aware of that examines the relationship between different definitions of symptom improvement and symptom remission and specifies empirically the percent change in symptoms associated with each of these outcome targets. High rates of clinical response to stimulant treatment in patients with ADHD have been well documented. This study identifies an empirical definition of percent symptom change that can be used as a standard to identify success in achieving optimal outcomes, and a way of differentiating small differences across trials that otherwise might not be apparent.

## Figures and Tables

**Table 1:** Baseline demographic and clinical characteristics (safety population, *n* = 230).

Characteristic, *n* (%)	MPH-MLR (*n* = 183)	Placebo (*n* = 47)
Age group, y		
6–8	49 (26.8)	11 (23.4)
9–11	56 (30.6)	20 (42.6)
12–14	55 (30.1)	8 (17.0)
15–18	23 (12.6)	8 (17.0)
Female	59 (32.2)	17 (36.2)
White	125 (68.3)	33 (70.2)
ADHD diagnosis subtype		
Combined	111 (60.7)	29 (61.7)
Predominantly inattentive	62 (33.9)	13 (27.7)
Predominantly hyperactive/impulsive	5 (2.7)	1 (2.1)
Not reported	5 (2.7)	4 (8.5)

ADHD: attention-deficit/hyperactivity disorder; MPH-MLR: methylphenidate extended release.
